# Unveiling common responses of *Medicago truncatula* to appropriate and inappropriate rust species

**DOI:** 10.3389/fpls.2014.00618

**Published:** 2014-11-07

**Authors:** Maria Carlota Vaz Patto, Diego Rubiales

**Affiliations:** ^1^Instituto de Tecnologia Química e Biológica António Xavier, Universidade Nova de LisboaOeiras, Portugal; ^2^Institute for Sustainable Agriculture, Consejo Superior de Investigaciones CientíficasCórdoba, Spain

**Keywords:** disease resistance, *Medicago truncatula*, nonhost, rust, *Uromyces*

## Abstract

Little is known about the nature of effective defense mechanisms in legumes to pathogens of remotely related plant species. Some rust species are among pathogens with broad host range causing dramatic losses in various crop plants. To understand and compare the different host and nonhost resistance (NHR) responses of legume species against rusts, we characterized the reaction of the model legume *Medicago truncatula* to one appropriate (*Uromyces striatus*) and two inappropriate (*U. viciae-fabae* and *U. lupinicolus*) rusts. We found that similar pre and post-haustorial mechanisms of resistance appear to be operative in *M. truncatula* against appropriate and inappropriate rust fungus. The appropriate *U. striatus* germinated better on *M. truncatula* accessions then the inappropriate *U. viciae-fabae* and *U. lupinicolus*, but once germinated, germ tubes of the three rusts had a similar level of success in finding stomata and forming an appressoria over a stoma. However, responses to different inappropriate rust species also showed some specificity, suggesting a combination of non-specific and specific responses underlying this legume NHR to rust fungi. Further genetic and expression analysis studies will contribute to the development of the necessary molecular tools to use the present information on host and NHR mechanisms to breed for broad-spectrum resistance to rust in legume species.

## INTRODUCTION

Rusts are a large group of obligate biotrophic basidiomycete fungi that can cause dramatic losses in various crop plants. Breeding for genetic resistance is the best measure for crop protection, because chemical control can have negative environmental effects and/or high economic costs.

For decades, selection for resistance to rust infection was based on highly specific, clearly recognized complete resistance, which is usually controlled by single genes (Ayliffe et al., 2008). This form of resistance provided, in most of the cases, only transient protection due to the evolution of virulent fungal isolates that negated breeders’ efforts and lead to spectacular “boom and bust” cycles. This has raised a major concern on durability of resistance and its implications for resistance breeding. It is today widely accepted that resistances genetically more complex, based on multiple loci, have the potential to be more durable. Nevertheless, durability is not only dependent on a complex genetic basis, but also on the resistance mechanisms involved, as some single-gene controlled mechanisms have proven to be more durable than others (Niks and Rubiales, 2002). The single-gene resistance most commonly used in rust resistance breeding is typically due to a post-haustorial defense mechanism, in which the plant cell collapses after the rust fungus started to form a haustorium in the cell resulting in hypersensitivity.

Although rarely exploited in breeding, mechanisms of resistance acting before the formation of haustoria also exist and contribute to increase the diversity of defenses to rust fungi. Indeed, rust infection can be hampered at very early stages of fungal development, from spore deposition to stomata recognition, resulting in a reduced penetration of the fungus into the tissue. Haustorium formation can be prevented by papilla deposition within plant cells attacked by haustorial mother cells. This type of resistance is very common in nonhost interactions. However, it can also be a significant component in host interactions, being important in the typically polygenic so-called partial resistance (Parlevliet, 1979), or in single gene-resistances that proved to be durable. Such examples are the *mlo* or *er1* resistances to powdery mildews (Jorgensen, 1992; Fondevilla et al., 2006), or the *Lr34* or *Lr46* resistance to leaf rust (Rubiales and Niks, 1995; Singh et al., 1998; Martínez et al., 2001).

Nonhost resistance (NHR) is the most common and robust situation in nature as most plant species are nonhosts of most pathogens (Lipka et al., 2010). The stability of NHR is attributed to multiple successive layers of protective mechanisms (preformed and induced) and a complex genetic control (Nürnberger and Lipka, 2005; Schulze-Lefert and Panstruga, 2011; Uma et al., 2011; Fan and Doerner, 2012). It is commonly speculated that NHR could be exploited by plants breeders seeking to improve disease resistance also within host species (Heath, 2000; Mysore and Ryu, 2004; Fan and Doerner, 2012).

Whereas induced defenses contribute to resistance to pathogens of plants of whatever close relationship with the nonhost (Niks, 2014), preformed barriers are more likely to contribute to NHR to pathogens of other plant families, than to pathogens of related plant species (Niks and Marcel, 2009). Little is known about the nature of effective defense mechanisms and respective genetic control in legumes to pathogens of remotely related plant species, especially rust pathogens with economic and biological importance (Cheng et al., 2012). Just a few studies have been performed on NHR to rust in legume species. Examples are *Medicago truncatula* to the Asian soybean rust, *Phakopsora pachyrhizi*, and to *Puccinia emaculata* (Uppalapati et al., 2012), faba bean (*Vicia faba*) to *P. striiformis* (Cheng et al., 2012), or common bean (*Phaseolus vulgaris*) to cowpea rust (*Uromyces vignae*; Heath, 1979).

In order to understand and compare the different host and NHR responses of a legume species against rusts, in this study we compared the model legume *M. truncatula* reaction to appropriate and inappropriate rusts inoculation.

## MATERIALS AND METHODS

### PLANT AND FUNGAL MATERIAL

Ten *M. truncatula* accessions [SA4327, SA9357, SA19995, SA21302, SA22182, SA25654, SA27778, SA28889, SA29831, SA30302, from the Australian Medicago Genetic Resource (SARDI)], differing in the level of resistance to *U. striatus*, selected based on a resistance screening performed earlier (Rubiales and Moral, 2004), plus two *M. truncatula* (Parabinga and Paraggio) and two alfalfa (*M. sativa*; Baraka and Ampurdam) susceptible cultivars were used to study the cellular responses to infection by three different *Uromyces* species. These were alfalfa rust, *U. striatus* (appropriate pathogen to *M. truncatula*) collected on alfalfa, in 2002, in Ubrique, Spain; faba bean rust, *U. viciae-fabae* collected on faba bean, in 2001, in Córdoba, Spain; and lupin rust, *U. lupinicolus* collected on lupin, in 2000, in Aberystwyth, UK (both inappropriate pathogens to *M. truncatula*). Monopustular isolates were stored in liquid nitrogen and multiplied before use on very susceptible cultivars of their proper hosts (*V. faba* cv. Baraca, *Lupinus albus* cv. Arthur, *M. sativa* cv. Baraka).

### INOCULATION AND INCUBATION

Seedlings were inoculated when the third trifoliate leaf was completely expanded. The leaf surface was inoculated by dusting 1 mg of freshly collected urediospores per plant, diluted in pure talc (1:10), resulting in a spore deposition of approximately 300 spores/cm^2^.

Plants were incubated for 24 h at 20°C in complete darkness and 100% relative humidity, and then transferred to a growth chamber at 20°C under a 14 h light: 10 h dark photoperiod, with light intensity of 148 μmol/m^2^/s at the leaf canopy. Each accession was represented by five seedlings in each rust isolate inoculation.

### HISTOLOGICAL OBSERVATIONS

Leaves were collected 1 d.a.i. (days after inoculation) and processed to study the phases of the fungus growth prior to stoma penetration, and 2 d.a.i to study the early stages of infection (Sillero and Rubiales, 2002), and the presence of necrosis. Three leaflet samples per seedling, per investigated time after inoculation, were cut.

The leaflet samples from 1 d.a.i. were laid, adaxial surface up, on filter paper dipped in fixative (1:1, absolute ethanol/glacial acetic acid, v/v). When the leaflet segments had been bleached by several changes of the fixative, they were transferred to filter paper moistened with tap water for at least 2 h, to soften the tissues. Next they were transferred to lactoglycerol (1:1:1, lactic acid/glycerol/water, v/v/v) for at least 2 h. To stain the samples, a drop of Trypan blue in lactoglycerol (0.1%, w/v) was placed on a cover glass; the sample was carefully laid with the adaxial surface toward the cover glass and then mounted in lactoglycerol on a microscope slide.

At 1 d.a.i., about 100 urediospores per leaflet sample were counted under 200 × magnification with a Leica DM LS microscope and grouped into the following categories: germinated urediospores (a spore was considered germinated when a germ tube at least as long as the diameter of the spore was produced); germ tubes growing over stomata, but not forming appressoria and germ tubes forming appressoria. Of the germ tubes which formed appressoria, distinction was made whether the appressorium was formed over a stoma or away from the stoma (misplaced).

The leaflet samples at 2 d.a.i were stained with Trypan blue (Sillero and Rubiales, 2002). Leaflets were fixed in acetic acid: ethanol (1:3 v/v) for 30 min; stained by boiling in 0.05% Trypan blue in lactophenol: ethanol (1:2 v/v) for 10 min and cleared in a nearly saturated aqueous solution of chloral hydrate (5:2 w/v) to remove Trypan blue from the chloroplast.

At 2 d.a.i., 30 random colonies/leaflet samples were studied. Numbers of hyphal tips and haustoria were recorded for each colony, along with the presence or absence of necrosis of plant cells associated to an infection structure.

### STATISTICAL ANALYSIS

Analysis of variance and comparison of means (Duncan test, *P* < 0.05) were performed for all microscopical components of resistance among accessions. When components of resistance were expressed as percentage, data were angular transformed prior to analysis of variance.

## RESULTS

In relation to the early stages of infection, *M. sativa* control accessions were, in general, not significantly different from *M. truncatula* controls against the three inoculated rust species. Exception was the reaction against *U. lupinicolus*, where all the *M. sativa* accessions presented significantly lower appressoria formation over a stoma than the *M. truncatula* lines (**Table 1**). In particular, the *M. truncatula* cv. Parabinga was amongst the accessions with lower appressoria formation over stoma by *U. striatus* and *U. viciae-fabae*, in the latter case together with the *M. sativa* cv. Baraka.

**Table 1:** *Uromyces striatus*, *U. viciae-fabae,* and *U. lupinicolus* growth prior to stoma penetration (1 d.a.i.) on selected *Medicago truncatula* accessions and alfalfa checks*.

Accession	*U. striatus*				*U. viciae-fabae*				*U. lupinicolus*			
	% germination	% lost germ tube	% misplaced appres.	% appres. over a stoma	% germination	% lost germ tube	% misplaced appres.	% appres. over a stoma	% germination	% lost germ tube	% misplaced appres.	% appres. over a stoma
*M. sativa* cv. Ampurdam	82.9^f^	55.1^bcd^	33.2^a^	41.5^cde^	47.7^cde^	63.5	9.7^bcde^	29.0^ab^	55.6	54.5^cd^	1.2^a^	20.6^ab^
*M. sativa* cv. Baraka	71.5^bc^	59.3^cd^	31.5^a^	40.2^cde^	42.6^abcd^	57.3	13.2^cdef^	26.7^a^	54.2	66.0^e^	5.3^b^	16.4^a^
*M. truncatula* cv. Parabinga	72.8^bcd^	74.6^ef^	44.2^b^	25.1^ab^	34.5^ab^	67.9	11.7^bcdef^	27.3^a^	46.5	53.5^bcd^	1.9^a^	38.6^cde^
*M. truncatula* cv. Paraggio	80.7^ef^	62.8^de^	33.0^a^	36.6^bcd^	41.2^abcd^	59.1	3.5^a^	38.9^bc^	51.5	58.8^de^	0.0^a^	35.2^cde^
SA4327	69.0^ab^	73.5^ef^	31.1^a^	24.9^ab^	44.2^bcd^	60.5	18.3^f^	31.3^abc^	49.7	46.5^abc^	1.8^a^	31.9^cd^
SA9357	73.0^bcd^	42.6^ab^	25.7^a^	55.2^fg^	44.4^bcd^	60.4	8.9^bcd^	35.8^abc^	45.6	41.8^a^	0.5^a^	46.6^e^
SA19995	75.7^bcde^	45.8^ab^	32.9^a^	48.3^defg^	44.1^bcd^	67.2	28.9^g^	26.1^a^	50.8	42.0^ab^	1.5^a^	41.2^de^
SA21302	75.6^bcde^	62.8^de^	30.0^a^	36.9^bcd^	50.9^de^	61.0	15.3^ef^	35.4^abc^	48.4	53.8^bcd^	0.7^a^	37.3^cde^
SA22182	77.2^cde^	47.6^abc^	28.8^a^	49.6^defg^	57.6^e^	67.1	19.1^f^	29.3^ab^	53.9	46.9^abcd^	0.8^a^	39.5^cde^
SA25654	78.3^cdef^	66.2^def^	32.7^a^	33.6^abc^	43.2^abcd^	60.6	18.0^f^	33.5^abc^	53.7	49.1^abcd^	2.7^a^	40.9^cde^
SA27778	64.4^a^	75.0^f^	45.2^b^	23.6^a^	41.3^abcd^	59.0	6.0^ab^	38.4^bc^	40.6	56.8^cde^	1.1^a^	35.8^cde^
SA28889	79.4^def^	37.2^a^	31.5^a^	58.8^g^	33.2^a^	69.4	8.2^abc^	27.4^a^	51.5	55.5^cde^	5.3^b^	34.2^cde^
SA29831	80.3^ef^	42.8^ab^	29.3^a^	52.2^efg^	44.1^bcd^	55.9	14.0^def^	40.1^c^	55.5	66.1^e^	9.5b	28.3^bc^
SA30302	75.1^bcde^	54.5^bcd^	32.7^a^	43.8^cdef^	39.7^abc^	62.3	13.0^cdef^	34.6^abc^	49.6	46.5^abc^	1.5^a^	45.2^de^
Selected *Mt* Average	74.8	54.8	32.0	42.7	44.3	62.3	15.0	33.2	49.9	50.5	2.5	38.1
ANOVA *p*	0.000	0.000	0.004	0.000	0.000	0.098	0.000	0.010	0.104	0.000	0.000	0.000

*Letters in common per column indicate that the values are not significantly different (*p* < 0.05, Duncan test). apress., appressoria.

Percentage of spore germination on *M. truncatula* accessions was higher for the appropriate rust *U. striatus* (74.8%) than for the inappropriate *U. viciae-fabae* (44.3%) and *U. lupinicolus* (49.9%). Little, although significant, genotypic differences were detected among *M. truncatula* accessions for the percentage of *U. striatus* and *U. viciae-fabae* germination, but not for *U. lupinicolus* (**Table 1**).

A similar proportion of germ tubes failed to orientate and find a stoma for the three rusts, with an average of 54.8% for *U. striatus*, 62.3% for *U. viciae-fabae* and 50.5% for *U. lupinicolus*. Significant genotypic differences were identified among *M. truncatula* accessions in the case of *U. striatus* infection, with values ranging from 37.2 to 74.6% of lost germ tubes. Significant, although smaller differences in percentage of lost germ tubes (in the range 42.0–66.1%), were also identified for *U. lupinicolus*, but not for *U. viciae-fabae*.

In all the *M. truncatula*/rust combinations studied, a proportion of germ tubes formed an appressorium away from the stoma. This proportion of so-called misplaced appressoria was relatively high for *U. striatus* (32%), with significant genotypic differences among *M. truncatula* accessions (in the range of 25.7–45.2%). Appressoria misplacement was intermediate for *U. viciae-fabae* (15%) and smaller for *U. lupinicolus* (2.5%), with significant genotypic differences among *M. truncatula* accessions detected in both cases. On average, a similar proportion of germ tubes successfully formed an appressoria over a stoma in all the three rusts, with averages of 42.7, 33.2, and 38.1% for *U. striatus*, *U. viciae-fabae,* and *U. lupinicolus*, respectively. Significant genotypic differences were identified among *M. truncatula* accessions in all the three rusts. Nevertheless, ranking in successful appressoria formation was not maintained across accessions for the three studied rusts. For instance, the highest appressoria formation over stoma of *U. striatus* and *U. viciae-fabae* (52.2 and 40.1%, respectively) was achieved on accession SA29831 that was, however, the lowest for *U. lupinicolus* (28.3%).

Early abortion of rust infection structures (**Table 2**) on *M. truncatula* accessions was very high for the inappropriate *U. viciae-fabae* and *U. lupinicolus* (96.9 and 93.9%, respectively), being also very high on alfalfa checks. Additionally, early abortion was, on average among *M. truncatula* accessions, still relatively high for *U. striatus* (48.4%) compared with alfalfa checks (21.7–27.5%). Significant differences, on the percentage of early abortion of *U. striatus*, were detected among the selected *M. truncatula* accessions, ranging from 18.1 to 80.1%. High levels of early abortion in all the studied rusts were detected, especially in accessions SA4327, SA22182, and SA28889, but not particularly associated with extreme cases of necrosis. Exception among these three consistent accessions, was accession SA4327, presenting a moderately high level of *U. viciae-fabae* early abortion with necrosis (61.7%). Differences in early abortion were also significant among *M. truncatula* accessions for *U. viciae-fabae* (range 76.9–100%), but not for *U. lupinicolus*. In particular, accessions SA30302, SA29831 and the two *M. sativa* control cultivars presented high levels of *U. viciae-fabae* and *U. lupinicolus* early abortion with necrosis (this last one, not in the case of accession SA30302). High levels of necrosis were also recorded, on established colonies, in accessions SA21302 and Paraggio against *U. striatus* (54.8 and 61.2%, respectively) or in accession SA30302 against *U. lupinicolus* (66.7%) infection.

**Table 2:** Early stages of the infection by *U. striatus*, *U. viciae-fabae,* and *U. lupinicolus* after stomata penetration (2 d.a.i.) on selected *M. truncatula* accessions and alfalfa checks*.

Accession	*U. striatus*			*U. viciae-fabae*			*U. lupinicolus*		
	Early abortion (%)	Early abortion with necrosis (%)	Established colonies with necrosis (%)	Early abortion (%)	Early abortion with necrosis (%)	Established colonies with necrosis (%)	Early abortion (%)	Early abortion with necrosis (%)	Established colonies with necrosis (%)
*M. sativa* cv. Ampurdam	21.7^a^	18.3	5.0^ab^	100.0^c^	57.4^bcd^	–	86.1	61.6^de^	50.0
*M. sativa* cv. Baraka	27.5^ab^	45.6	32.3^abcd^	96.9^bc^	69.2^d^	0.0	100.0	74.8^e^	–
*M. truncatula* cv. Parabinga	32.2^ab^	1.7	0.0^a^	89.9^b^	6.3^a^	0.0	97.1	6.8^ab^	0.0
*M. truncatula* cv. Paraggio	34.4^ab^	27.0	61.2^d^	100.0^c^	38.1^abcd^	–	88.0	31.9^abcde^	42.9
SA4327	73.5^cd^	46.4	41.4^bcd^	100.0^c^	61.7^bcd^	–	100.0	44.6^bcde^	–
SA9357	36.2^ab^	25.6	13.2^abc^	98.8^c^	38.0^bcd^	0.0	98.1	27.6^abcd^	50.0
SA19995	na	na	na	76.9^a^	46.5^bcd^	0.0	72.0	14.3^abc^	6.4
SA21302	48.1^bc^	31.6	54.8^cd^	100.0^c^	37.4^bcd^	–	95.7	21.7^abcd^	50.0
SA22182	70.8^cd^	38.1	33.3^abcd^	100.0^c^	41.8^bcd^	–	95.8	25.0^abcd^	0.0
SA25654	18.1^a^	33.3	40.0^bcd^	100.0^c^	36.5^bcd^	–	90.1	40.7^bcde^	41.7
SA27778	23.6^ab^	25.0	42.3^bcd^	100.0^c^	25.8^abc^	–	100.0	3.0^a^	–
SA28889	80.1^d^	28.1	10.7^abcd^	100.0^c^	25.5^ab^	–	100.0	43.5^bcde^	–
SA29831	49.6^bc^	34.6	28.8^abcd^	93.3^bc^	70.0^cd^	0.0	93.1	58.8^cde^	25.0
SA30302	35.4^ab^	60.2	45.3^bcd^	100.0^c^	72.5^d^	–	93.9	26.7^abcd^	66.7
Selected *Mt* Average	48.4	32.3	31.0	96.9	45.6	0.0	93.9	30.6	34.2
ANOVA *p*	0.000	0.241	0.008	0.000	0.002	–	0.152	0.004	0.383

*Letters in common per column indicate that the values are not significantly different (*p* < 0.05, Duncan test). na, non-available.

On average, the few *U. viciae-fabae* colonies developed in the selected *M. truncatula* accessions, were bigger (higher number of hyphal tips and haustoria per established colony; 5.7 and 2.5) than the *U. striatus* (4.2 and 1.4) or *U. lupinicolus* (2.6 and 1.1) colonies. Some of the selected *M. truncatula* accessions (SA29831 and SA30302) showed bigger or equal size *U. striatus* colonies than the *M. sativa* controls (6.6 and 2.2; 4.8 and 1.6; respectively). *M. truncatula* accessions SA29831, SA19995, and cv. Parabinga had extremely big *U. vicia-fabae* colonies, and in addition, accession SA19995 and cv. Paraggio, big *U. lupinicolus* colonies (**Table 3**).

**Table 3:** Colony development by *U. striatus*, *U. viciae-fabae,* and *U. lupinicolus* (2 d.a.i.), on selected *Medicago truncatula* accessions and alfalfa checks*.

Accession	*U. striatus*		*U. viciae-fabae*		*U. lupinicolus*	
	N° hyphal tips/ established colony	N° haustoria established colony	N° hyphal tips/ established colony	N° haustoria/ established colony	N° hyphal tips/ established colony	N° haustoria/ established colony
*M. sativa* cv. Ampurdam	4.8^de^	1.8^cde^	-	-	3.5^bc^	1.0
*M. sativa* cv. Baraka	6.1^f^	2.1^e^	6.0^b^	3.0	-	-
*M. truncatula* cv. Parabinga	5.0^e^	2.0^de^	7.8^bc^	3.2	3.3^abc^	1.0
*M. truncatula* cv. Paraggio	4.5^cde^	1.7^bcde^	-	-	4.6^c^	1.3
SA4327	4.4^bcde^	1.4^abc^	-	-	-	-
SA9357	3.9^abcd^	1.6^abcde^	2.0^a^	1.0	2.0^ab^	1.0
SA19995	na	na	6.3^b^	2.6	4.6^c^	1.8
SA21302	3.7^abc^	1.5^abcd^	-	-	1.7^ab^	1.3
SA22182	4.2^bcde^	1.2^ab^	-	-	1.0^a^	1.0
SA25654	3.4^ab^	1.4^abc^	-	-	3.0^abc^	1.3
SA27778	4.0^abcde^	1.6^abcde^	-	-	-	-
SA28889	3.1^a^	1.1^a^	-	-	-	-
SA29831	4.8^de^	1.6^abcde^	9.0^c^	4.0	4.0^bc^	1.0
SA30302	6.6^f^	2.2^e^	-	-	2.0^ab^	1.5
Selected *Mt* Average	4.2	1.4	5.7	2.5	2.6	1.1
ANOVA *p*	0.000	0.000	0.000	0.127	0.000	0.246

*Letters in common per column indicate that the values are not significantly different (*p < 0.05*, Duncan test).

## DISCUSSION

*U. striatus* is an important fungal disease of worldwide distribution, being particularly damaging in alfalfa grown for seed (Leath et al., 1988). It has a broad host range comprising many species from the tribes Trifolieae, Cicereae, and Vicieae, including alfalfa and annual medics, such as the model *M. truncatula* (Skinner and Stuteville, 1995). The fact that *M. truncatula* is susceptible to *U. striatus* opens the way for its use to unravel legume-rust interactions.

A range of resistance mechanisms is operative in *M. truncatula* accessions against *U. striatus* (Rubiales and Moral, 2004; Kemen et al., 2005). Here we showed that similar mechanisms are operative against the inappropriate *U. viciae-fabae* and *U. lupinicolus*, although specific responses have been also detected against particular inappropriate rust species. NHR is the most common and durable form of resistance (Heath, 2000). Therefore the identification and incorporation of traits that confer NHR to a broad range of rust fungi is an attractive and durable alternative to host resistance breeding (Uppalapati et al., 2012). This study by identifying similarities between NHR to inappropriate pathogens and basal resistance to appropriate pathogens provides useful information for future broad spectrum resistance breeding.

The appropriate *U. striatus* germinated better on *M. truncatula* accessions than the inappropriate *U. viciae-fabae* and *U. lupinicolus*, but once germinated, germ tubes of the three rusts had a similar level of success in finding stomata and forming an appressoria over a stoma. This contrasts with what was reported in other legume, broad bean, NHR to rust, where the successful location of stomata by the inappropriate wheat stripe rust was significantly reduced (Cheng et al., 2012). However, similarly to most rust plant hosts (Niks and Rubiales, 2002), pre-penetration resistance mechanisms, including reduction of urediospore germination and fungal development on the leaf surface, seem to be of marginal importance in *M. truncatula* against *U. striatus*, at best, in reducing infection levels.

Early abortion of infection structures was however, on average, high for *U. striatus* compared with alfalfa checks, with significant variation across *M. truncatula* accessions. Moreover, for the inappropriate rusts, *U. viciae-fabae* and *U. lupinicolus*, this component of resistance was even of higher importance on all *M. truncatula* selected accessions, being associated or not, with host cell necrosis, depending on the genotype. Pre-haustorial or penetration resistance is common in nonhost interactions with haustorium forming specialized pathogens (Heath, 1974; Elmhirst and Heath, 1987; Niks, 1987), being typically associated with the formation of cell wall reinforcements, also called cell wall appositions or papillae (O’Connell and Panstruga, 2006). Pre-haustorial resistance can be also identified in host interactions, playing a major role on the so-called partial resistance, which most times is more durable than resistance controlled by R genes (Niks and Rubiales, 2002). Frequently, the pre-haustorial NHR is backed-up by a hypersensitive post-penetration resistance for those infection units that still succeed in cell wall penetration (Heath, 2002; Lipka et al., 2005). Indeed similar percentage of early abortion with associated necrosis was detected on all the selected *M. truncatula* accessions against the appropriate and the two inappropriate rusts evaluated. This was also the case in the broad bean/wheat stripe rust NHR reported by Cheng et al. (2012). Furthermore in our study clear genotypic differences in percentage of early abortion associated with necrosis were only detected amongst the *M. truncatula* reaction to inappropriate rusts. Nevertheless, necrosis was not associated with established colonies only in the case of *U. viciae-fabae*, resulting consequently in the bigger colony development of the three analyzed rust species. Genotypic differences have been in addition detected among the selected *M. truncatula* accessions in relation to some of the described pre and post-haustorial resistance components against appropriate, but also against inappropriate rust pathogens. Variation in the effectiveness of the NHR in several plant/rust combinations has been recently revised by Niks (2014), describing the existence of variable successful infection by rust fungi on a small proportion of nonhost plant species accessions. It has been proposed that NHR in plant may be also due to stacked R-genes of the NB-LRR type, varying their relative contribution as a function of the phylogenetic divergence time between host and nonhost (Schulze-Lefert and Panstruga, 2011). However, when there is no colocalization of NHR QTLs with known R genes or R-gene analogs or when NHR is of pre-haustorial type, this hypothesis does not stand (Niks and Marcel, 2009). As an example, little evidence was found for the involvement of R-genes in the genetic control of the microscopic variation in the effectiveness of the barley NHR to the heterologous grass and cereal rust fungi (Niks, 2014). According to this author, NHR of barley to rust fungi of related Gramineae results from the joined effect of multiple, quantitative genes (QTLs), although occasionally, a major gene might be involved. Most barley NHR QTLs to rust infection are pathogen species-specific, having effect to only one or two heterologous rusts, but some have a wider spectrum, suggesting a combination of non-specificity and specificity of the genes underlying NHR to rust fungi. This might be also the case in our studied *M. truncatula* accessions NHR to rust.

*Medicago truncatula* is being studied to unravel resistance to a broad number of pathogens using an increasing number of genomic tools (see Rubiales et al., 2011, for a review) that are contributing to enhance understanding of its interaction with rust both at genomic (Madrid et al., 2010) and proteomic level (Castillejo et al., 2010). Future genetic and expression analysis studies involving inappropriate rust inoculation in *M. truncatula* as the ones described here, may reveal if the same defense-related genes or QTLs are involved in host and NHR, and confirm if the similarities now detected on the phenotypic resistance components would stand at molecular level. These future studies might contribute to the development of the necessary molecular tools to breed for broad-spectrum resistance to rust in legume species.

We conclude that similar pre and post-haustorial mechanisms of resistance appear to be operative in *M. truncatula* against appropriate and inappropriate rust fungus. However, as already described for other plant/rust interactions (Niks, 2014), responses to different inappropriate rust species also show different particularities among *M. truncatula* accessions, suggesting a combination of non-specific and specific responses underlying this legume NHR to rust fungi.

## AUTHOR CONTRIBUTIONS

Maria Carlota Vaz Patto carried out the experiments and analyzed the data. Maria Carlota Vaz Patto and Diego Rubiales planned the study and wrote the manuscript. Both authors read and approved the final manuscript.

## Conflict of Interest Statement

The authors declare that the research was conducted in the absence of any commercial or financial relationships that could be construed as a potential conflict of interest.
